# SNP Array Karyotyping Allows for the Detection of Uniparental Disomy and Cryptic Chromosomal Abnormalities in MDS/MPD-U and MPD

**DOI:** 10.1371/journal.pone.0001225

**Published:** 2007-11-21

**Authors:** Lukasz P. Gondek, Andrew J. Dunbar, Hadrian Szpurka, Michael A. McDevitt, Jaroslaw P. Maciejewski

**Affiliations:** 1 Experimental Hematology and Hematopoiesis Section, Taussig Cancer Center, Cleveland Clinic, Cleveland, Ohio, United States of America; 2 Hematology, Internal Medicine, Hematological Malignancy, and Oncology, Johns Hopkins University School of Medicine and The Sidney Kimmel Cancer Center, Baltimore, Maryland, United States of America; Ordway Research Institute, United States of America

## Abstract

We applied single nucleotide polymorphism arrays (SNP-A) to study karyotypic abnormalities in patients with atypical myeloproliferative syndromes (MPD), including myeloproliferative/myelodysplastic syndrome overlap both positive and negative for the JAK2 *V617F* mutation and secondary acute myeloid leukemia (AML). In typical MPD cases (N = 8), which served as a control group, those with a homozygous *V617F* mutation showed clear uniparental disomy (UPD) of 9p using SNP-A. Consistent with possible genomic instability, in 19/30 MDS/MPD-U patients, we found additional lesions not identified by metaphase cytogenetics. In addition to UPD9p, we also have detected UPD affecting other chromosomes, including 1 (2/30), 11 (4/30), 12 (1/30) and 22 (1/30). Transformation to AML was observed in 8/30 patients. In 5 *V617F+* patients who progressed to AML, we show that SNP-A can allow for the detection of two modes of transformation: leukemic blasts evolving from either a wild-type *jak2* precursor carrying other acquired chromosomal defects, or from a *V617F+* mutant progenitor characterized by UPD9p. SNP-A-based detection of cryptic lesions in MDS/MPD-U may help explain the clinical heterogeneity of this disorder.

## Introduction

Acquired loss of heterozygosity (LOH) can occur either as a result of deletions or mitotic recombination (uniparental disomy [UPD]). LOH has been described in many malignant hematologic conditions including acute myelogenous leukemia (AML), as well as solid cancers [1]–[5]. Using 50K single nucleotide polymorphism arrays (SNP-A), we recently identified a high frequency of UPD in myelodysplastic syndromes (MDS) as well, occurring in approximately 30% of patients [6]. A similar result was subsequently found in low-risk MDS subtypes [7]. Moreover, other previously cryptic aberrations not detected by metaphase cytogenetics (MC) were identified in all sub-types of MDS, including secondary leukemias [6]. We have also extensively validated this technology with regard to its sensitivity and resolution for detecting acquired loss of heterozygosity, gains, and micro-deletions [8].

LOH can lead to decreased gene expression or hemi/homozygosity of germ line variants or somatic mutations affecting the remaining allele. One such example is JAK2 (*147796) *V617F*, a gain-of-function mutation often associated with myeloproliferative syndromes (MPD), particularly polycythemia vera [9]–[13]. Studies using microsatellite markers, as opposed to other methods such as MC or fluorescence *in situ* hybridization (FISH), which cannot detect LOH due to UPD, have shown that UPD9p leads to homozygosity of the *V617F* mutation [14]. The *V617F* mutation has been rarely found in de novo AML [15]–[18], but has recently been associated with MDS/MPD overlap syndromes and in some cases, AML evolving from MPD [19], [20].

The WHO classification of myeloid malignancies distinguishes MDS/MPD as a separate entity and includes chronic myelomonocytic leukemia (CMML), MDS/MPD-Unclassifiable (MDS/MPD-U), and refractory anemia with ringed sideroblasts and thrombocytosis (RARS-t) [21]. Analysis of a large cohort of these patients has revealed that the *V617F* mutation is present in only small proportion of these patients [20]. However, we have shown recently that patients with RARS-t have a higher incidence of *V617F* [22] suggesting that *V617F* genotyping and evaluation would be useful in classification and clinical evaluation.

In addition, larger chromosomal abnormalities as detected by metaphase cytogenetics are also common in patients with typical MPD or MDS/MPD [23]. For example, 46% patients with myelofibrosis demonstrated an abnormal metaphase karyotype involving interstitial deletions of the long arm of chromosomes 13 or 20, among others [24]. In addition, MPD is the underlying diagnosis in 5% of patients with 5q abnormalities [25], and in PV, 25.4% of patients showed clonal abnormalities. The recurrent chromosomal lesions were those of chromosome 9 (21.1%), del(20q) (19.2%), trisomy 8 (19.2%), rearrangements of 13q (13.4%), and abnormalities of 1q (11.5%), chromosome 5 and chromosome 7 (9.6%) [26].

We hypothesize that if cytogenetic methods with a higher resolution are used, additional defects, including UPD of chromosomal regions other than 9p, will be detected. Furthermore, we stipulate that SNP-A can allow for a convenient method of identifying chromosomal changes in patients who transform to AML.

To investigate these hypotheses, we have applied 250K SNP-arrays (SNP-A) to examine genomic composition and identify previously cryptic chromosomal defects and molecular abnormalities in a group of patients with MDS/MPD-U and secondary AML developed from MDS/MPD-U both positive and negative for the V617F mutation. We hypothesize that while UPD9p leads to homozygosity of the JAK2 mutation in MPD, other occurrences of UPD on additional chromosomes in MDS/MPD-U patients may contribute to the phenotypic features that give rise to this ambiguous disease.

## Results

### Detection of UPD9p in patients with MPD as a demonstration of SNP-A to identify copy-neutral LOH

First, we performed SNP-A karyotyping in 58 control marrows. Normal copy number polymorphisms were easily detected and excluded from the patient analysis along with any others previously reported in the literature or on available internet databases [27]. LOH was detected in 4/58 healthy controls and tended to be smaller in size. Consequently, for the analysis of any patients, these changes were not deemed significant and similar to other lesions, were excluded from the analysis. Repeated analysis of samples (N = 6) showed high congruency (data not shown) and analysis of SNP calls spanning chromosome X in males revealed remarkable fidelity with an accuracy rate of >99% [8].

**Figure 1:**
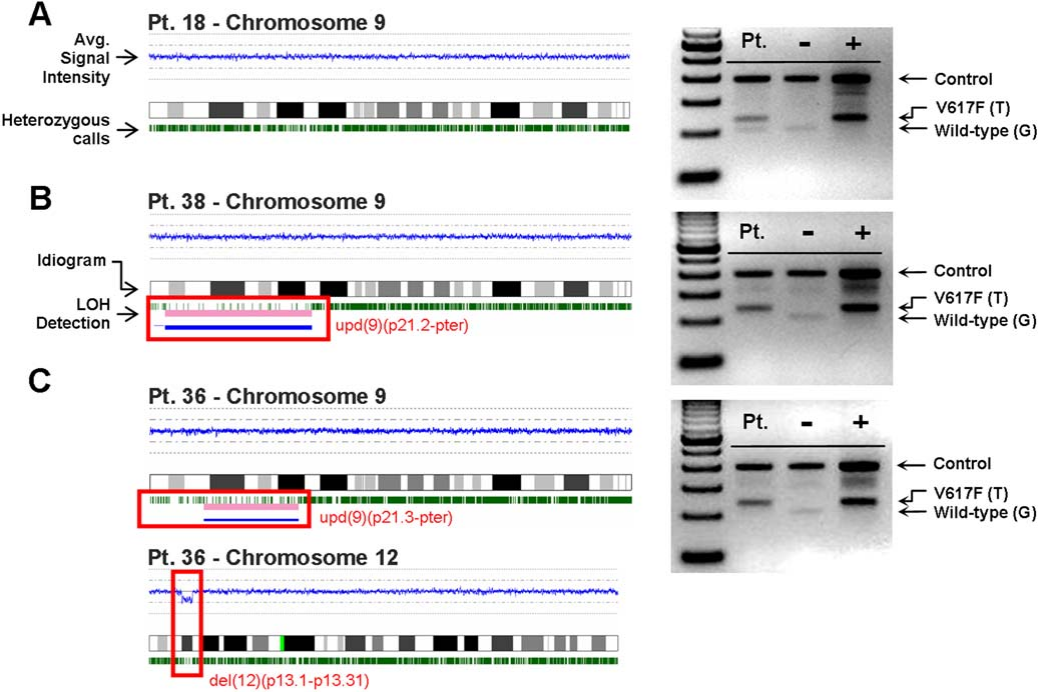
SNP karyograms confirm loss of heterozygosity in patients homozygous for JAK2 V617F. SNP-A based karyotypic analysis on chromosome 9 for (A) a patient heterozygous for the JAK2 V617F mutation and (B,C) two patients homozygous for the JAK2 V617F mutation. (A,B and C, left portion) Signal intensity and SNP karyograms for each corresponding patient; the blue line represents the average fluorescent signal intensity of each SNP and oscillates around the diploid marker line; green tics represent heterozygous calls for each individual SNP. Areas of UPD are associated with the absence of heterozygous calls and are highlighted by blue and pink bars. Extraneous calls in regions of UPD occur as a result of contamination by non-clonal cells. UPD was confirmed by microsatellite analysis (data not shown). (C) In addition to chromosome 9, patient #36 also exhibited a segmental deletion in chromosome 12 as indicated by decreases in the copy number and frequency of heterozygous calls. (A,B, and C, right portion) Corresponding ARMS-PCR analysis of the JAK2 V617F mutation in each patient confirms heterozygous (A) and homozygous (B,C) mutational status (gel images are cropped and enhanced).

To determine whether or not SNP-A can be used as an accurate and effective method to identify acquired UPD, we studied patients with typical MPD homozygous for the *V617F* mutation. In 4 control patients homozygous for *V617F* (Pts. #34, #36, #37 and #38, **Tables 1** **&** **2**), UPD9p was easily detected by SNP-A; in addition, those positive control patients heterozygous for the *V617F* mutation showed normal diploid copy number by SNP-A (except for Pt. #31 with trisomy 9), and therefore lacked any UPD9p (**Figure 1**). However, it is possible that an abnormal clone harboring UPD9p exists in heterozygous V617F patients but is too small to be detected using current methods. Regardless, similar to results recently published [8], [28], we have shown that SNP-A provides a simple and effective tool for detecting acquired UPD.

**Table 1:** Patients participating in the study.

	Pt.	Age	Sex	Initial Diagnosis	Trans. to AML (Y/N)	Clinical features	JAK2 V617F Genotype
MDS/MPD (N = 30)	1	65	F	MDS/MPDu	N	Increased small megakaryocytes in clusters, pSTAT5+ staining in megakaryocytes, fibrosis	G/G
	2	60	M	MDS/MPDu	N	Left-shifted leukocytosis, leukoerythroblastic changes, dyspalstic erythroid series, dysplastic megakaryocytes, fibrosis	G/G
	3	76	M	MDS/MPDu	N	Increased dysplastic megakaryocytes, megaloblastoid dyserythropoiesis, dysgranulopoiesis with hypogranular PMNs, fibrosis, increased mast cells	G/G
	4	77	M	MDS/MPDu	N	Increased dysplastic megakaryocytes, absent erythropoiesis, left-shifted granulopoiesis, dysgranulopoiesis	G/G
	5	65	F	MDS/MPDu	N	Increased dysplastic megakaryocytes, fibrosis	G/G
	6	73	M	MDS/MPDu	N	Mild to moderate dyserythopoiesis, fibrosis	G/G
	7	62	F	RARSt	N	Dyserythropoiesis and ringed sideroblasts, dysplastic megakaryocytes	G/G
	8	72	M	MDS/MPDu	N	Increased dysplastic megakaryocytes in clusters, marked fibrosis	G/G
	9	41	F	MDS/MPDu	N	Dyserythropoiesis, left-shifted, dysplastic granulopoiesis	G/G
	10	80	M	RARSt	N	Increased ringed sideroblasts, dyserythropoiesis, fibrosis, pSTAT5+ staining in erythroid precursors and megakaryocytes, thrombocytosis	G/G
	11	67	M	MDS/MPDu	N	Dysplastic megakaryocytes, dysgranulopoiesis, fibrosis	G/G
	12	76	F	MDS/MPDu	N	Dysplastic megakaryocytes, dyserythropoiesis, dysgranulopoiesis, fibrosis	G/G
	13	73	M	MDS/MPDu	N	Dysplastic megakaryocytes, fibrosis	G/G
	14	73	M	RARSt	N	Dysplastic megakaryocytes in clusters, ringed syderoblasts, fibrosis, thrombocytosis	G/G
	15	74	F	RARSt	N	Dysmegakaryopoiesis, ringed sideroblasts, increased proerythroblasts, thrombocytosis	G/G
	16	60	M	RARSt	N	Increased dysplastic megakaryocytes, numerous ringed sideroblasts, fibrosis, thrombocytosis	G/T
	17	60	F	RARSt	N	Increased ring sideroblasts, dysmegakaryopoiesis, thrombocytosis	G/T
	18	76	M	RARSt	N	Increased ringed sideroblasts, increased dyplastic large megakaryocytes, megaloblastic erythropoiesis with mild megaloblastoid change, fibrosis, thrombocytosis	G/T
	19	75	F	RARSt	N	Megaloblastoid dyserythropoiesis, increased ringed sideroblasts, dysmegakaryopoiesis, fibrosis, thrombocytosis	G/T
	20	79	M	MDS/MPDu	N	Left shifted hyperplastic dysgranulopoiesis, increased dysplastic megakaryocytes, fibrosis, monocytosis	G/T
	21	70	M	MDS/MPDu	N	Dysplastic megakaryocytes, splenomegaly	G/T
	22	76	F	RARSt	N	leukocytosis with absolute monocytosis, thrombocytosis, numerous ringed sideroblasts, fibrosis	G/T
	23	67	F	MDS/MPDu	Y	Erythroid dsplasia, dysplastic granulopoiesis, increased megakaryocytes, fibrosis	G/G
	24	71	M	MDS/MPDu	Y	Megaloblastic erythropoiesis, left-shifted dysplastic granulopoiesis, dysplastic megakaryocytes, fibrosis, hepatosplenomegaly	G/G
	25	80	F	MDS/MPDu	Y	Dysplastic granulocytopoiesis, megaloblastoid and dysplastic erythroid maturation, dysplastic megakaryocytes	G/G
	26	73	M	MDS/MPDu	Y	Increased dysplastic megakaryocytes, dyserythropoiesis, fibrosis	G/T
	27	72	F	MDS/MPDu	Y	Dysplasia involving all lineages, fibrosis, increased megakaryocytes	G/T
	28	54	M	MDS/MPDu	Y	Increased dysplastic megakaryocytes in clusters, fibrosis	T/T
	29	65	M	MDS/MPDu	Y	Left-shifted, leukocytosis, normocytic anemia, thrombocytopenia, splenomegaly	T/T
	30	62	F	MDS/MPDu	Y	Increased dysplastic megakaryocytes in clusters, fibrosis	T/T
MPD (N = 8)	31	76	F	PV	N	Polycythemia	G/T
	32	78	M	PV	N	Polycythemia	G/T
	33	81	F	PV	N	Polycythemia	G/T
	34	62	M	PV	Y	Polycythemia	T/T
	35	63	F	PV/IMF	N	Fibrosis, polycythemia, leukoerythroblastic changes	G/T
	36	67	M	IMF	N	Fibrosis, splenomegaly, leukoerythroblastic changes	T/T
	37	56	M	IMF	Y	Fibrosis, splenomegaly, leukoerythroblastic changes	T/T
	38	77	M	IMF	Y	Fibrosis, splenomegaly, leukoerythroblastic changes	T/T

MDS/MPD-U: myeloproliferative disorder/myelodysplastic syndrome overlap, unclassifiable; RARSt: refractory anemia with ringed sideroblasts and thrombocytosis; PV: polycythemia vera; IMF: idiopathic myelofibrosis,

**Table 2:** Cryptic chromosomal abnormalities identified in patients with MDS/MPD-U and secondary AML with and without the JAK2 V617F mutation.

	Pt.	Lesions detected by Metaphase Cytogenetics (MC)	Additional Lesions found by SNP-A (New lesions not detected by MC)
MDS/MPD	1	46,XX,del(5)(q12q33)[cp7]	NAL
	2	46,XY[20]	add (16)(q23.1)
	3	46,XY[20]	NAL
	4	46,XY,del(7)(q11.2),del(20)(q11q13),-21,+r[cp20]	del(7)(p12.3-p14.1), del(7)(q11.22-qter), del(11)(p15.4-p15.5), add(21)(complex), UPD(22)(q11.21-qter)
	5	46,XX[20]	add(1)(p32.2), add(X)(p22.31)
	6	46,XY[20]	add(2)(p16.1), del(6)(q16.1)
	7	46,XX[20]	NAL
	8	46,XY,+8[9]/46,XY[11]	UPD(6)(p21.32-p22.2)
	9	46,XX,t(6;9)(p23;q34)[20]	UPD(11)(q14.1-q14.2), UPD(12)(p11.21-p12)
	10	46,XY[20]	NAL
	11	46,XY,del(20)(q11.2q13.3)[19]/46,XY[1]	del(11)(q14.1), del(12)(p13.1-p13.31)
	12	46,XX,del(4)(p14),del(5)(q13q33),del(12)(q23),del(13)(q14q22),I(17)(q10)[8]/46,idem,t(2;7)(p12;q36)[10]/47,XX,del(4)(p14),del(5)(q13q33),add(11)(p15),add(12)(p13),del(13)(q14q22),I(17)(q10),+mar[2]	del(2)(p26.1-p26.2), del(4)(p15.1), del(7)(q34), del(11)(p15.4-pter), del(12)(q24.31), del(13)(q22.1-q22.2), del(13)(q33.1), del(17)(p13.1), del(21)(q21.2-q22.11)
	13	46,XY[11]	UPD(3)(p23-p24.1), UPD(3)(q12.3-q13.12), UPD(11)(q13.4-qter)
	14	46,XY[20]	NAL
	15	47,XX,+8[4]/46,XX,[16]	NAL
	16	46,XY[20]	NAL
	17	46,XY, del(5)(q) [20]	NAL
	18	46,XY[20]	NAL
	19	46,XX,inv(9)(p11q12)[20]	UPD(1)(p11.2-pter), del(2)(p16.2), del(22)(q11.23)
	20	46,XY [10]; 47,XY,+8,[9]; 46,XY,del(20)(q11.2) [1]	NAL
	21	46,XY,del(2)(p22),inv(9)(p12q13),del(20)(q12)[4]	del(8)(q11.23-q12.1), add(9)(p12-pter)
	22	46,XX[20]	NAL
	23	46,XX,der(3)(3pter->3q13.1::3q21->3q21::3q24->3qter),+8[22]	del(3)(q13.1-q21.3), del(9)(p23), UPD(11)(q12.2-q13.3)
	24	48,XY,+8[6]/46,XY[14]	UPD(1)(q25.2-25.3)
	25	46,XX[20]	UPD(11)(p), del(16)(p12.1)
	26	47,XY,+8[14]	del(2)(q23.3-q24.1), del(4)(q26.1), del(6)(q23.2-q23.3), +13, +17q, -17p*
	27	44-45,XX,del(5)(q13q33),-6,-10,-13, add(14)(q32), add(17)(p11.2),add(20)(p11.2),+r,+mar[cp3]/46,XX[6]	del(9)(p21.1)
	28	47,XY,+8[12]/46,XY[8]	UPD(9)(p13.2-pter)
	29	46,XY,der(6)t(1;6)(q25;p23)[14]/46,XY[6]	del(4)(q24), UPD(9)(p21.1-pter)
	30	47,XY,del(3)(q21q26.2),+5,del(5)(q32)x2,add(7)(q22), del(7)(q?32),inv(12)(q13q15)[20]	add(3)(q21.3), del(3)(q21.3), add(5)(q13.3-pter), del(5)(q13.3), del(5)(q31.2), del(7)(q22.1), del(7)(q34), del(7)(q36.1), UPD(9)(p13.3-pter), del(10)(p12.1)
MPD	31	49,XX,+8,+9,+21[8]/46,XX[12]	NAL
	32	N/A	del(13)(q12.3-q31.1)
	33	46,XX[20]	del(18)(q12.1-q12.2)
	34	46,XY[20]	UPD(7)(q22.1-qter), UPD(9)(p13.3-pter)
	35	N/A	del(1)(p36.21-p36.32), del(5)(q21.3-q33.3), del(9)(p13.2-p21.2), del(9)(q21.13-q21.33), del(9)(q22.31-q31.1)
	36	46,XY[20]	UPD(9)(p21.3-pter), del(12)(p13.1-p13.31)*
	37	46,XX,del(13)(q12q22)[15]/46,XX[5]	UPD(9)(p13.3-pter), UPD(11)(q12.3-qter), del(13)(q13.2-q31.1)
	38	46,XY[20]	UPD(9)(p21.2-pter)*

NOTE: copy number lesions identified by MC were confirmed by SNP-A. Any significant changes in the size of regions initially reported by MC are noted in the right-hand column along with other additional lesions found by SNP-A.

Abbreviations: N/A: no aspirate obtained; NAL: no additional lesions found; *SNP-A data obtained using granulocyte DNA.

### Detection of cryptic chromosomal abnormalities in MDS/MPD-U patients

Using SNP-A, clonal lesions, including segmental UPD, were found in 23/30 (77%) patients as compared to 18/30 (60%) by conventional metaphase cytogenetics (**Table 2**). All unbalanced copy number changes found by MC were confirmed by SNP-A, and in most instances, allowed for further refinement to more isolated regions. UPD, including UPD9p, appears to be a common chromosomal defect, occurring on chromosomes other than 9 in 8/30 patients (i.e. on chromosomes 1, 11, and 22) (**Table 3**). In addition, other copy number changes were detected as well, including segmental micro-deletions of chromosomes 1, 5, 9 and 12, among others (**Table 2**). Any shared/overlapping lesions found in 3 or more patients were isolated and are indicated in Table 4. Not surprisingly, the most common region of overlap was that of 9p spanning the region of the *jak2* gene. Likewise, other common lesions often associated with these diseases were identified, including del5q and trisomy 8. However, three previously cryptic overlapping regions shared in 3 or more patients were identified (**Table 4**). These regions consisted of three small segmental deletions of chromosome 7 (7q22.1, 7q34, and 7q36.1), a small cytoband (q14.1) of chromosome 11 (as defined by patients #9, #11 and #13), and small sub-sections of the q arm of chromosome 20 lying within the area of cytobands q11.23 to q12 (as defined by patients #4, #11, #20, and #21).

**Table 3:** UPD found in MDS/MPD-U patients both positive and negative for the JAK2 V617F mutation.

V617F+		V617F-	
*UPD*	*N*	*UPD*	*N*
UPD(1)	1	UPD(1)	1
UPD(9)	3	UPD(3)	1
		UPD(6)	1
		UPD(11)	4
		UPD(12)	1
		UPD(22)	1

**Table 4:** List of commonly deleted regions in MDS/MPD-U patients.

Lesion	N
del(13)(q14-q22)	3
del(7)(q22.1)/del(7)(q34)/del(7)(q36.1)	3
del/UPD(11)(q14.1)	3
del/UPD(11)(p15.4-p15.5)	3
del(20)(q11.2-q12)	4
del(5)(q13-q33)	5
add/UPD(9)(p13.3-pter)	4
Trisomy 8	7

Analysis of patients with MDS/MPD-U who progressed to AML compared to those with a stable course of the disease showed, as expected, a greater number of lesions detected in the first group (8/8 vs. 15/22). Within cohorts studied, Kaplan-Meier analysis of survival shows no difference between patients with or without previously cryptic defects uncovered by SNP arrays (data not shown).

### Comparison of chromosomal abnormalities in *V617F+* and *V617F-* MDS/MPD-U

We have identified patients (**Tables 1** **&** **2**) with a history of MDS/MPD-U both positive (N = 12) and negative (N = 18) for the JAK2 *V617F* mutation and have analyzed them using 250K SNP-A. Conventional MC revealed chromosomal aberrations in 9/12 (75%) of those positive for the mutation, including common abnormalities such as +8 and del5q. A majority of the *V617F+* MDS/MPD-U patients (7/9) with abnormal MC showed previously undetected additional lesions, including UPD on chromosomes other than 9 in only 1/12 patients (**Table 2**). Examples of deleted regions in patients positive for the mutation include segmental losses within chromosomes 2, 4, 5 and 20 and UPD on chromosomes 1p and 9p. However, these micro-deletions and instances of UPD appear to be highly variable from patient to patient with no substantial overlap occurring between more than two patients.

For those MDS/MPD-U patients negative for the *V617F* mutation, previously undetected lesions were also identified by SNP-A. Of these 18 patients, 9 showed abnormal MC. However, when SNP-A was applied, 5 additional patients with normal MC were found to have lesions, and 7 of the 9 patients with abnormal MC had lesions in addition to those detected by MC. Examples of these lesions include micro-deletions of chromosomes 6 and 20. UPD was more common in *V617F-* patients, occurring in 7/18 patients, predominantly on chromosome 11 (in 4 of the 7 patients).

### Detection of cryptic chromosomal abnormalities in patients with secondary AML evolving from JAK2 *V617F+* disease

**Figure 2:**
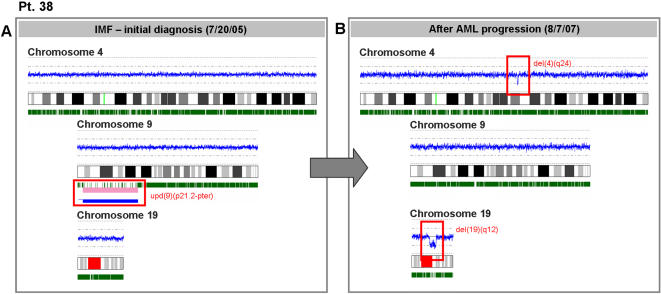
SNP-A can be used to identify lesions acquired during AML evolution. SNP-A karyograms demonstrate that before transformation (A), patient #38 showed only UPD9p at initial diagnosis as a sole abnormality (consistent with a homozygous JAK2 V617F mutation) along with normal chromosomes 4 and 19. However, after transformation to AML (B), repeated SNP-A analysis showed the presence of a V617F- leukemic clone with a normal chromosome 9 and newly-acquired micro-deletions on both chromosomes 4 and 19.

**Figure 3:**
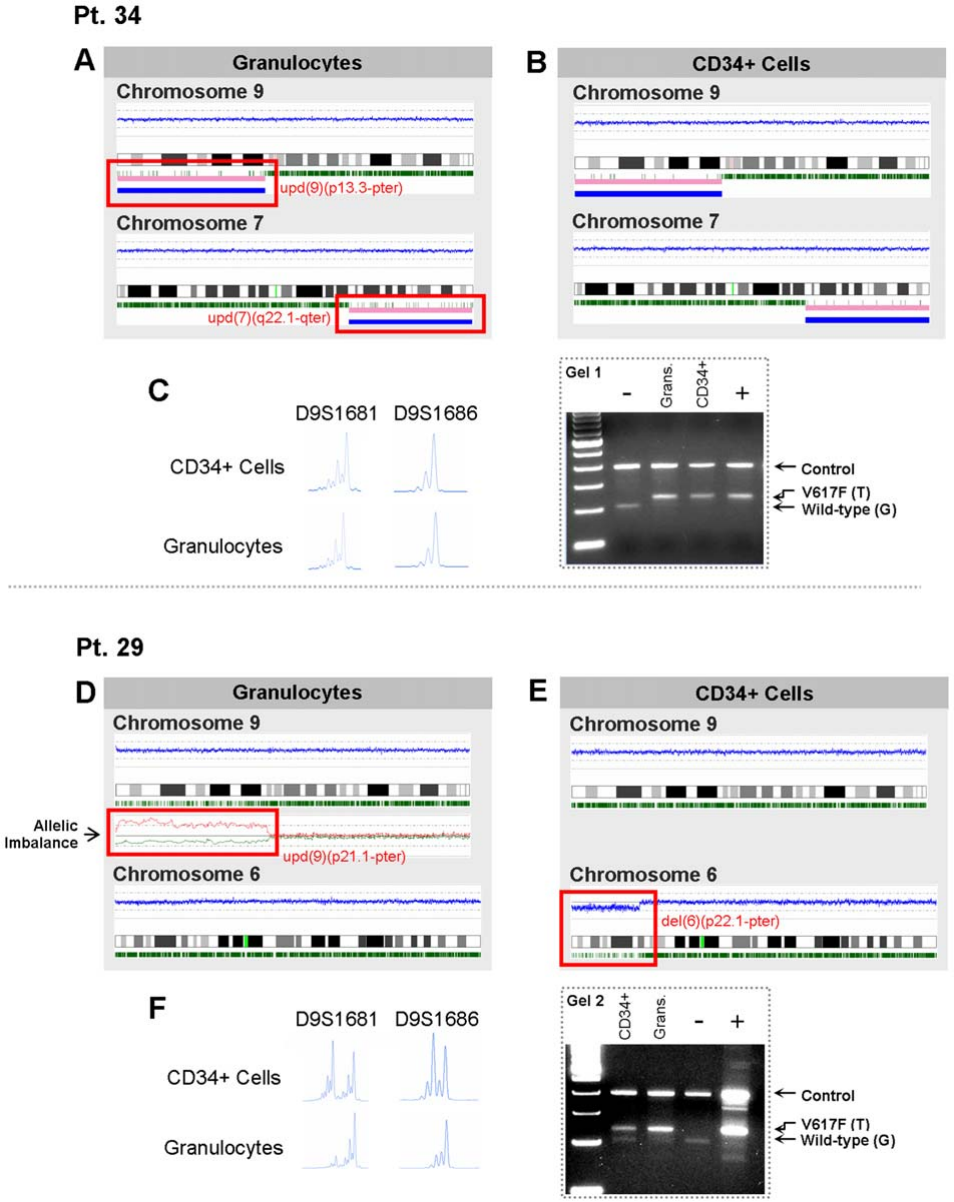
Karyotypic analysis of AML evolution in patients with the JAK2 V617F mutation. Karyograms illustrate the contributing lesions in patient #34 (A, B) and patient #29 (D, E). In panels C and F, microsatellite analysis confirms the UPD for patients #34 and #29, respectively. In patient #34 (A,B,C), only one distinct clone was identified: in both granulocytes (A) and blasts (B), loss of heterozygosity calls on chromosome 9 confirms UPD9p, consistent with the homozygous JAK2 V617F mutation. In addition, both cell types also possess UPD on 7q. (C, left portion) Microsatellite analysis of blasts and granulocytes confirms LOH on 9p in both cell types. (C, right portion) Electrophoresis gel of ARMS-PCR in patient #34 also shows a homozygous JAK2 V617F mutation in both granulocytes and blasts (gel image is cropped and enhanced). In patient #29 (D,E,F), two distinct clones were identified using DNA isolated from both granulocytes and CD34+ blasts. In granulocytes (D), UPD9p is consistent with a homozygous JAK2 V617F mutation while analysis of chromosome 6 did not reveal any abnormalities. LOH on chromosome 9 was not completely resolved in the karyogram obtained; however, comparative analysis of granulocytes and lymphocytes confirmed allelic imbalance (red line: lymphocytes, green line: granulocytes). In contrast, when CD34- selected blasts were analyzed (E), UPD9p was not identified while a segmental deletion on chromosome 6 was found. In panel F, microsatellite analysis and electrophoresis gel of ARMS-PCR demonstrate the presence of homozygosity for the JAK2 V617F mutation in granulocytes but heterozygosity in CD34+ cells.

SNP-A can also be used to identify lesions acquired during AML evolution. We investigated cytogenetic implications of AML transformation in 5 patients. In 2 patients with classical MPD (#34 and #38), SNP-A showed two distinct modes of transformation to AML. In patient #38, UPD9p was present at diagnosis, consistent with a homozygous JAK2 *V617F* mutation. However, upon transformation to AML, repeated SNP-A analysis now showed the presence of a new, *V617F-* leukemic clone with a normal chromosome 9 and previously absent micro-deletions on both chromosomes 4 and 19 (**Figure 2**). In the second patient, transformation was accompanied by the presence of UPD9p (consistent with homozygous Jak2 mutation) and UPD7q. Both lesions were present in blasts as well as in mature granulocytes suggesting an evolution from within the JAK2 mutated clone (**Figure 3A, B, C**).

We performed similar analysis of 3 MDS/MPD-U patients who transformed to AML (**Tables 1** **&** **2**; patients #26, #28 and #29). Similar to patient #38 above and in accordance with recent published findings [29], [30], patient #26 showed two distinct clones upon transformation; one contributing to mature cell production characterized by the *V617F* mutation and a second with wild-type *jak2* (data not shown). However, in 2 of 3 MDS/MPD-U patients (Pts. #28 and #29), we identified the presence of a clone possessing both the JAK2 *V617F* mutation and additional chromosomal lesion(s) associated with AML transformation. This result was obtained by comparison of SNP-A data from both granulocytes and sorted CD34+ blasts (**Figure 3D, E, F**). More interestingly, patient #29 seems to harbor 2 clones: one clone with UPD9p that resulted in a homozygous JAK mutation and an AML clone which possesses 1 mutated and 1 wild type *jak2* allele. Granulocyte idiograms demonstrated the presence of UPD9p (confirming the homozygous *V617F* clone) and an otherwise normal chromosome set. In CD34+ blasts, however, a normal chromosome 9 was present but a new del6p was detected, likely contributing to leukemic transformation. ARMS-PCR (**Figure 3F**, right panel) showed a mutated *jak2* (T) and wild-type allele (G) in the blast fraction suggesting that the AML clone arose from a cell heterozygous for the *jak2* mutation.

## Discussion

Some cases of MPD show a pathophysiologic overlap with MDS [21], [22], [31], and in some instances, the JAK2 mutation is present. A significant clinical heterogeneity, ranging from common chronic course to less frequent evolution to AML [13], [16]–[18], exists among classical MPD patients with the JAK2 *V617F* mutation, and is even more diverse in patients with MDS/MPD overlap. It is possible that some of this variability in clinical phenotype may be related to the diverse spectrum of cytogenetic defects found in these patients. The presence of these cytogenetic abnormalities, as detected by SNP-A, supports the theory that MPD patients have an underlying propensity to chromosomal breaks and subclonal evolution. This feature may be particularly accentuated in patients with MDS/MPD-U overlap, although only a minority of these patients, perhaps with exception of RARS-t, harbor the *V617F* mutation [20], [22].

We have compared the results of MC and SNP-A karyotyping in patients with MDS/MPD-U with and without the JAK2 *V617F* mutation. SNP-A allowed for the detection of previously cryptic chromosomal abnormalities in 19/30 patients with MDS/MPD-U, including those who showed normal MC. In addition to gains and deletions, SNP-A allowed for efficient detection of UPD. The most illustrative example of this type is UPD9p itself, which was found in all patients homozygous for the *V617F* mutation. Recently, the utility of SNP-A for identification of UPD9p was demonstrated in both MPD [28] and MDS [7], [8] and our results show that UPD due to mitotic recombination can affect not only the p-arm of chromosome 9 but also other regions of the genome. While there was an abundance of UPD found in these patients, no significant overlap occurred, perhaps due to the size of the cohort studied. However, one region of overlap, located on chromosome 11q14.1, harbors genes of particular interest, including *rab30* (*605693), a proto-oncogenic member of the RAS family, and perhaps most notably, *gab2* (*606203), which encodes an adaptor protein that mediates the interaction between STAT5 and phosphatidylinositol-3 kinase (PI3k) in activation of the PI3k/Akt pathway [32], [33].

Copy-neutral loss of heterozygosity has been described in several malignancies [1]–[5] but due to the inability of MC to identify UPD, it has remained undetected in many patients. Previously, we have demonstrated that UPD is quite prevalent in patients with MDS and can be found in approximately 30% of cases studied by either 50K or 250K SNP-A [8].

In general, there was no difference in the types of lesions present in patients with and without the *V617F* mutation; however, UPD was present more often in patients who were negative for the mutation. As expected, UPD9p was not encountered in patients either wild-type or heterozygous for the JAK2 mutation.

In the course of our study, we have observed a number of patients who progressed from MDS/MPD-U and MPD to AML. SNP-A helped to delineate the origin of the AML clone. The pathogenesis of AML in patient #26 and #38 is consistent with previous reports which show that in a majority of cases, transformation to AML in patients with classical MPD occurs in a stem cell with a wild-type *jak2* gene [29], [30]. However, we have found that in some instances (pts. #28, #29, and #34), AML does originate from a stem cell with a mutant *jak2* gene. The transformation process is often associated with the acquisition of additional defects (e.g. del6p as demonstrated in patient #29, or micro-deletions of chromosomes 4 and 19 as demonstrated in patient #38).

Our ability to detect cryptic lesions is related to the technical advantages of SNP-A-based karyotyping, which allows for the identification of smaller lesions and copy number neutral changes (UPD); however, one drawback to SNP-A karyotyping is its inability to recognize balanced translocations [34].

In order to assess the significance of clonal chromosomal lesions present in patients with MDS/MPD-U, study of normal control specimens is essential. We have found a large number of known copy number polymorphisms detected in controls, but UPD was only found in 4/58 patients and tended to be much smaller than that seen in patients. For the purpose of our study, all copy number changes seen in patients were compared to those described both in public databases and those present in our own control group. Subsequently, all copy number variations found in normal controls were eliminated from our patient samples and analysis. In previous studies, we have extensively validated the results obtained by 50K and 250K arrays using FISH, MC, microsatellite PCR, and TaqMan PCR for copy number determination [6], [8]. However, the sensitivity of SNP-A for the number of clonal cells in the sample is limited: as tumor content decreases, LOH detection rate steeply declines, and with <20–30% tumor cells, no LOH can be detected, even when complete genotype information for both tumor and paired constitutive DNA is obtained [28].

In summary, our results demonstrate the applicability of SNP-A-based karyotyping for detecting clonal cytogenetic abnormalities in MDS/MPD-U. This new technology allows for precise definition of chromosomal aberrations, including copy-neutral LOH, and complements MC in detecting chromosomal lesions in MDS/MPD-U. Our results demonstrate that UPD is a common form of LOH in both JAK2 *V617F+* and *V617F-* disorders and is not restricted only to chromosome 9p but can affect other regions which may potentially point towards causative genes.

## Materials and Methods

### Patients

Bone marrow aspirates were collected from patients with MDS/MPD-U (N = 30) and MPD (N = 8) (mean age 69 years; range 41–81) seen between 2002–2007. Patients were grouped according to the World Health Organization (WHO) classification system [35] (**Table 1**). For the purpose of the study, we have focused our analysis on patients with MDS/MPD. This includes MDS/MPD-U, RARS-t, CMML and atypical CML. However, CMML and atypical CML represent a phenotypically distinct subtype of MDS/MPD and have not been included in this study. In 5 patients who transformed to AML we studied both original clones as well as leukemic blasts. Informed consent for sample collection was obtained according to protocols approved by the Cleveland Clinic IRB. Aspirates obtained from 58 healthy individuals (mean age 43 years; range 27–61) were used as controls.

### Cytogenetic analysis

Cytogenetic analysis was performed on marrow aspirates according to standard methods. Chromosome preparations were G-banded using trypsin and Giemsa (GTG), and karyotypes were described according to ISCN [36].

### Cell separation

In some experiments, granulocytes were used as a source of DNA for analysis and were isolated during density centrifugation separation from pelleted cells. For separation of CD34+ cells and CD3+ lymphocytes, blood or marrow mononuclear cells were isolated using Ficoll density gradient centrifugation and then separated using magnetic beads (StemCell Technologies, Vancouver, Canda) on the RoboSep Instrument (StemCell Technologies) according to the protocol provided by the manufacturer. The purity of isolated cells was >95% as measured by flow cytometry (Coulter Elite, Hielah, FL).

### DNA extraction

DNA was extracted from whole bone marrow with the ArchivePure DNA Blood Kit (5Prime, Gaithersburg, MD, USA). Red blood cell lysis solution was added to whole bone marrow at a 3∶1 ratio and incubated with shaking for 10 minutes. The cells were pelleted and the DNA extracted as per the manufacturer's instructions. The concentration of the DNA was obtained using a ND-1000 spectrophotometer (NanoDrop, Wilmington, DE, USA) and the quality determined by gel electrophoresis.

### Allele-specific polymerase chain reaction (PCR)

The status of the JAK2 V617F mutation was determined by a DNA tetra-primer ARMS assay as previously described [37]. The sequences of the primers were as follows: 5′-GCATTTGGTTTTAAATTATGGAGTATATG-3′, 5′-GTTTTACTTACTCTCGTCTCCACAAAA-3′, 5′-AAGCACATTGTATCCTCATCTATAGTCA-3, 5′-GAATAGTCCTACAGTGTTTTCAGTTTCA-3′. Wild-type *G/G* genotype generates 2 bands on gel electrophoresis at 379bp and 201bp, homozygous *T/T*: 2 bands at 379 bp and 234 bp, and heterozygous *G/T*: all 3 bands. Gels were photographed, cropped, and enhanced using the Bio-Rad Gel Doc XR machine and Quantity One 4.5.2 software (Bio-Rad, Hercules, CA).

### SNP-A analysis

The Gene Chip Mapping 250K Assay Kit (Affymetrix, Santa Clara, CA) was used for SNP-A analysis. Following Nsp I digestion, fragmented DNA was ligated to adaptor followed by PCR amplification. The PCR product was hybridized to the GeneChip Mapping 250K Nsp Array, processed with the Fluidic Station and the Gene Chip Scanner 3000 (Affymetrix). Only DNA samples with a call rate of >90% were used. To determine the minimal clonal size that can be detected by SNP-A, dilution studies of trisomy 21 DNA with normal diploid DNA were performed; clones spanning more than 25–50% of the total cell population can be detected. Unlike comparative genomic hybridization, SNP-A does not require reference DNA as it obtains copy number and loss of heterozygosity calls through analysis of hybridization frequencies using probes designed to detect individual SNP alleles [34].

### Microsatellite and gene copy number (CN) analysis

In general, SNP-A as a karyotyping platform was validated in our previous studies with regard to sensitivity, resolution, and ability to detect clonal vs. germ line chromosomal defects [8], [34]. When appropriate, regions of LOH for this study were confirmed by microsatellite (MS) polymorphism analysis. Primer sequences were obtained from the NCBI database (http://www.ncbi.nlm.nih.gov). Forward primers were modified at the 5′ end with FAM fluorescent dye. DNA was amplified and amplicons analyzed using ABI Prism 310 Genetic Analyzer (Applied Biosystems, Foster City, CA). CN analysis was performed by microsatellite analysis using a Real-Time TaqMan chemistry protocol [38]. The probe for detection of CA repeats was designed as a 21-bp oligomer containing GT repeats with FAM and Black Hole Quencher modifications on 5′ and 3′ ends, respectively. All reactions were performed in triplicate using the D12S1699 amplicon as an endogenous control.

### Biostatistical evaluation

Signal intensity was analyzed and SNP calls determined using Gene Chip Genotyping Analysis Software Version 4.0 (GTYPE). CN was investigated using a Hidden Markov Model and CN Analyzer for Affymetrix GeneChip Mapping 250K arrays (CNAG 2.0) [39]. Segmental LOH was identified by a statistical assessment of the likelihood that consecutive SNP loci would exhibit heterozygosity given the corresponding allelic frequency of particular SNP in the normal population (CNAG). The two-sided Fisher's Exact test was used to analyze the difference between the distribution of dichotomized variables among the groups.
